# Synthesis and Properties of the La_1 − *x* − *y*_Eu_*y*_Ca_*x*_VO_4_ (0 ≤ *x*, *y* ≤ 0.2) Compounds

**DOI:** 10.1186/s11671-017-2116-7

**Published:** 2017-05-08

**Authors:** O. V. Chukova, S. G. Nedilko, A. A. Slepets, S. A. Nedilko, T. A. Voitenko

**Affiliations:** 1 Faculty of Physics, Taras Shevchenko National University of Kyiv, 64/13, Volodymyrska Str, 01601 Kyiv, Ukraine; 2 Faculty of Chemistry, Taras Shevchenko National University of Kyiv, 64/13, Volodymyrska Str, 01601 Kyiv, Ukraine

**Keywords:** Vanadate, Sol–gel, Eu^3+^, Luminescence

## Abstract

The La_1 − *x*_Ca_*x*_VO_4_ and La_1 − *x* − *y*_Eu_*y*_Ca_*x*_VO_4_ (0 ≤ *x*, *y* ≤ 0.2) micro/nanosized powders were prepared by aqueous nitrate–citrate sol–gel synthesis. Phase composition of the sample depends on the *x* and *y* values. The La_0.9_Ca_0.1_VO_4_ is crystallized in monoclinic structure up to the *x* = 0.1. The La_0.9_Eu_0.05_Ca_0.05_VO_4_ sample was also attributed to the monoclinic structure. Increasing concentration of europium and calcium ions in La_1 − *x* − y_Eu_*y*_Ca_*x*_VO_4_ solid solutions leads to the change of the crystal structure, and subsequently, stabilization of the tetragonal phase takes place.

The obtained samples were characterized by XRD analysis, SEM microscopy, and IR spectroscopy. Luminescence properties of the synthesized powders were studied. Emission of the La_1 − *x*_Ca_*x*_VO_4_ samples is weak and consists of wide bands in the 450–800 nm spectral range. The observed bands at 570 and 630 were ascribed to electron transitions in the distorted VO_4_^3−^ vanadate groups. Emission of the La_1 − *x* − *y*_Eu_*y*_Ca_*x*_VO_4_ samples consists of narrow spectral lines in the 550–730 nm spectral range. The lines are caused by the ^5^D_0_ → ^7^F_J_ electron transitions in the Eu^3+^ ions. The Ca^2+^ ions incorporation increases the intensity of the Eu^3+^ ions luminescence. Structure of the spectra depends on Ca^2+^ concentration and excitation wave length. The carried out analysis has revealed that Eu^3+^ ions form at least two different types of emission centers in the La_1 − *x* − *y*_Eu_*y*_Ca_*x*_VO_4_ samples. The assumption is made that type I centers are formed by the Eu^3+^ ions in their regular positions in the crystal lattice, while the type II centers have complex structure and consist of Eu^3+^ ions, Ca^2+^ cations, and oxygen vacancies.

## Background

Rare earth orthovanadates are very interesting class of compounds which have very important applications in various fields involving chemistry and biology, luminescent nanoparticles, and light transformers [1–4]. Orthovanadates can exhibit unusual magnetic, optical, thermally activated, and X-ray luminescence properties [5]. Nanosized orthovanadates have also attracted considerable research interest as perspective photocatalyst systems for photocatalytic water splitting. The widely used photocatalyst TiO_2_ is active only under ultraviolet light irradiation [1, 6], while europium (EuVO_4_) [7] and lanthanum (LaVO_4_) [8] orthovanadates are of a special attention as the most promising visible-light-driven photocatalysts [9]. These compounds are chemically stable and non-toxic [10, 11]. Their range of biological applications includes fluorescent probes for single-molecule tracking, drug development, protein detection, and fluorescent bio labeling [2, 11, 12].

Actually, some of the abovementioned applications require vanadate materials with improved efficiency of luminescence excitation under light from near UV and violet spectral ranges [4, 13–15]. The search for new vanadate compounds for these needs is carried out using variations of two and more cations in their composition including partial iso- and heterovalent substitutions [16–19]. It was shown previously that intensities of luminescent emission of the RE activators in orthovanadate compounds can be effectively increased with the A^2+^ modifying cations (A = Ca, Sr, Ba, Pb, etc.) [4, 16, 20–22]. Moreover, properties of compositions with such heterovalent substitutions are strongly dependent on concentration ratios of the A^2+^ cations. Besides, our recent investigations have shown that increase of intensity of luminescence emission of rare earth orthovanadates can be also achieved with improvement of method of synthesis and morphology of the nanoparticles [14, 23]. Therefore, we expect that expansion of the best of our recent practices for synthesis of the rare earth orthovanadate nanaparticles onto the same compositions with heterovalent substitutions could bring an additional raise of emission intensity of luminescent orthovanadate nanoparticles. At the first step in this direction, we use the Ca^2+^ modifying impurities as the cheapest reagent.

The aim of this work is to study synthesis procedures, structural features, and morphological and optical characteristics of the nanosized La_1 − *x*_Ca_*x*_VO_4_ and La_1 − *x* − *y*_Eu_*y*_Ca_*x*_VO_4_ (0 ≤ *x*, *y* ≤ 0.2) compounds. Various methods can be applied for synthesis of the orthovanadates nanoparticles, such as solid state [16, 23], hydrothermal [24], solution combustion [17], and sol–gel methods [14, 25]. One of the most promising ways to perform an excellent homogenization for nanoscale sizes of particles, high reactivity of the compounds, and morphology that satisfy enhanced emission intensity is the sol–gel method.

## Methods/Experimental

### Synthesis

**Fig. 1:**
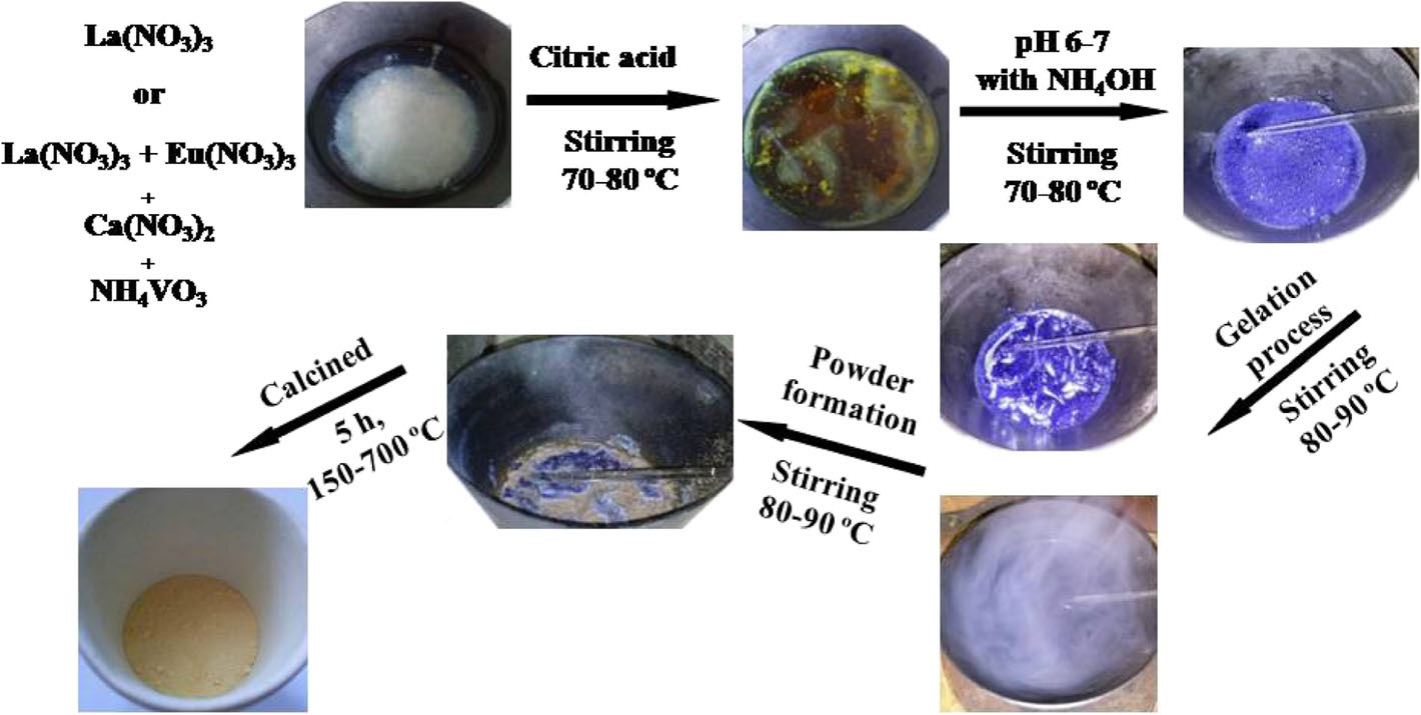
Schematic diagram showing the experimental process for synthesis of the La_1 − *x*_Ca_*x*_VO_4_ and La_1 − *x* − *y*_ Eu_*y*_Ca_*x*_VO_4_ (0 ≤ *x*, *y* ≤ 0.2) compounds

The La_1 − *x*_Ca_*x*_VO_4_ and La_1 − *x* − *y*_Eu_*y*_Ca_*x*_VO_4_ samples (0 ≤ *x*, *y* ≤ 0.2) were prepared by aqueous nitrate–citrate sol–gel synthesis route using citric acid (CA) as a complexing agent. Lanthanum (III) and europium (III) nitrates La(NO_3_)_3_ (99.0%, High Purity Chemicals), Eu(NO_3_)_3_ (99.0%, High Purity Chemicals), calcium (II) nitrate Ca(NO_3_)_2_ (99.0%, High Purity Chemicals), and ammonium metavanadate NH_4_VO_3_ (99.0%, High Purity Chemicals) were used as starting compounds. They were weighted according to the desired stoichiometric molar ratio. Nitric acid (HNO_3_), distilled water, and ammonia (NH_3_·4H_2_O) were used as solvents and reagents to regulate the pH of the solutions. Firstly, NH_4_VO_3_ was dissolved in concentrated ammonia solution by stirring at 70–80 °C temperature. Then, the CA dissolved in distilled water with a small amount of NH_3_·4H_2_O was added. Next, La(NO_3_)_3_, Eu(NO_3_)_3_, and Ca(NO_3_)_2_ were added. To prevent precipitation, the pH that reached the value of ~6–7 was controlled. Finally, the same amount of the aqueous solution of the complexing agent CA was repeatedly added to the reaction mixture to prevent crystallization of metal salts during the gelation process. The clear solution was concentrated by slow evaporation at 80–90 °C in an open beaker. A transparent gel has been formed after evaporation of nearly 90% of the water during the continuous stirring. The powders were obtained after drying in an oven at 100 °C. The powders were step by step calcined for 5 h at the range from 150 to 700 °C. Figure 1 illustrates the process of synthesis.

### Equipment

Phase compositions and crystal lattice parameters of the synthesized samples were determined using X-ray diffractometer DRON-3M (CuKα-radiation with a Ni filter). The diffraction patterns were taken with a 2°/min step. Characterization of the samples’ morphology was made using scanning electron microscope (SEM) Tescan Mira 3 LMU with 20-nm electronic beam diameter. The secondary electron detector (InBeam) could be used, if there is a need, to enhance spatial resolution up to 1 nm. Infrared (IR) spectra of the samples were recorded on PerkinElmer IR spectrometer using the KBr pellet method in the 1400–400 cm^−1^ range. Luminescence spectra were measured at high resolution equipment using DFS-12 and DMR-23 diffraction spectrometers with excitation by diode lasers, nitrogen gas laser, and xenon lamp (see more details in [26, 27]).

## Results and Discussion

### X-ray Diffraction

**Fig. 2:**
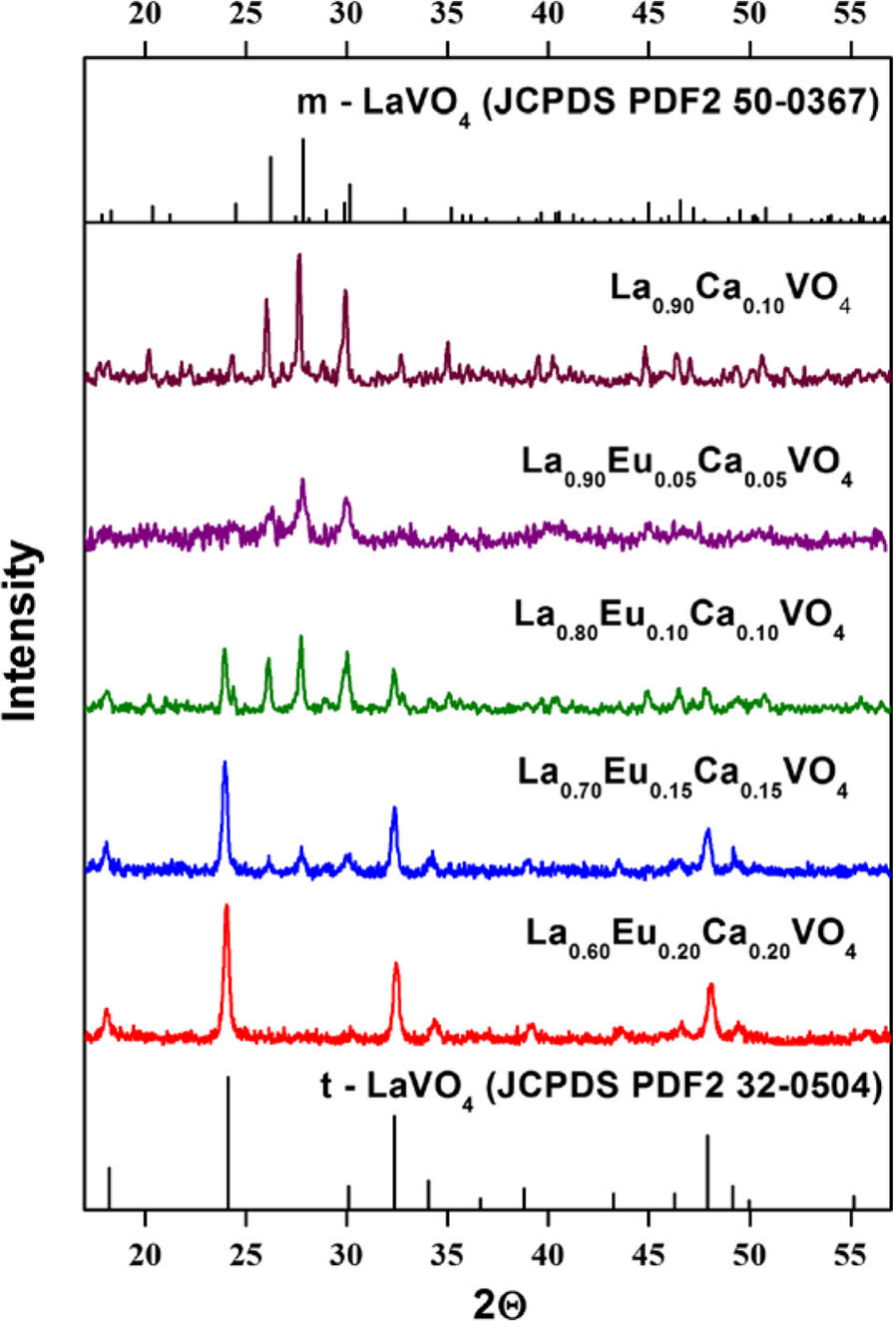
X-ray diffraction patterns of the La_1 − *x*_Ca_*x*_VO_4_ and La_1 − *x* − *y*_Eu_*y*_Ca_*x*_VO_4_ (0 ≤ *x*, *y* 0.2) samples

The X-ray diffraction (XRD) patterns of the synthesized La_1 − *x*_Ca _−_ VO_4_ and La_1 − *x* − *y*_Eu_*y*_Ca_*x*_VO_4_ (*x* ≤ 0.2, *y* ≤ 0.2) samples are shown in Fig. 2. It was found that the La_0.9_Ca_0.1_VO_4_ sample is crystallized in monoclinic structure; a space group is P2_1_/n (see Fig. 2). This result is well matched with a standard card of monoclinic LaVO_4_ (JCPDS PDF2 50-0367) (see Fig. 2). The La_0.9_Eu_0.05_Ca_0.05_VO_4_ sample with low concentration of the Ca^2+^ and Eu^3+^ impurities is also attributed to the monoclinic structure. Proximity of the Ca^2+^ (1.12 nm), La^3+^ (1.16 nm), and Eu^3+^ (1.066 nm) ions radii provides an opportunity for calcium and europium ions’ incorporation into three plus cation’s sites. Besides, we see that small quantities of the Ca^2+^ (*x* ≤ 0.1) and Eu^3+^ ions (*y* ≤ 0.05) can enter to the crystal lattice without change of the monoclinic LaVO_4_ structure.

Increasing concentrations of the Eu^3+^ and Ca^2+^ impurities leads to formation of the monoclinic and tetragonal phase mixture (Fig. 2). The La_0.6_Eu_0.2_Ca_0.2_VO_4_ sample becomes related to tetragonal structure, belonging to the I41/amd space group. This result is agreed with the standard card of tetragonal LaVO_4_ (JCPDS PDF2 32-0504) (see Fig. 2). If some amount of the residual monoclinic phase is still presented in these samples, it was below the detection limit of our XRD equipment.

Therefore, increasing concentration of europium and calcium ions in La_1 − *x* − *y*_Eu_*y*_Ca_*x*_VO_4_ (*x*, *y* ≤ 0.2) solid solutions leads to the change of the crystal structure, and subsequently, stabilization of the tetragonal phase takes place.

### Morphology and Chemical Element Analysis

**Fig. 3:**
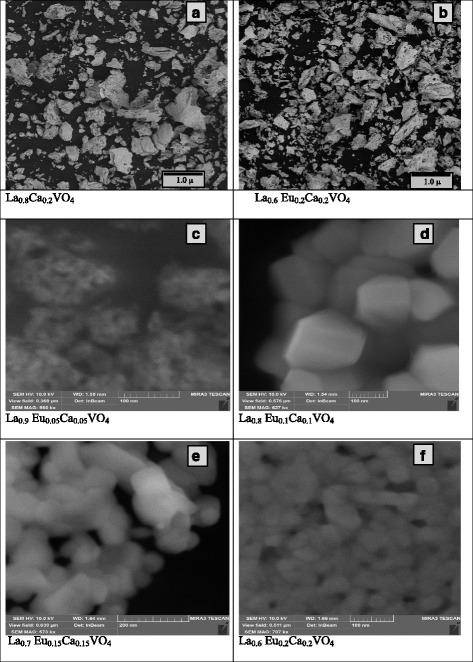
The SEM images of the La0.8Ca0.2VO4 (**a**), La0.6 Eu0.2Ca0.2VO4 (**b**, **f**), La0.9 Eu0.05Ca0.05VO4 (**c**), La0.8 Eu0.1Ca0.1VO4 (**d**), and La0.7 Eu0.15Ca0.15VO4 (**e**) samples

The set of characteristic SEM images of the La_1 − *x* − *y*_Eu_*x*_Ca_*y*_VO_4_ samples are shown in Fig. 3. The top two images, a and b, show areas of ~5 × 5 μm; in other words, general view of samples is given, whereas other images, c–f, show small separated areas, up to ~0.5 × 0.5 μm size. It is easy to see from general view that samples consist of grains of different sizes, from tens of nanometers to 1 μm. Grains of large sizes (0.2–1.0 μm) are agglomerates of smaller ones, and they have no certain shapes. Detailed view of agglomerates and of small grains (Fig. 3c–f) show that they are formed by nanoparticles of size from 20 to 100 nm. The La_0.9_Eu_0.05_Ca_0.05_VO_4_ samples with low concentration of the Eu and Ca impurities consist of grains of 0.1–0.2 μm-size, those are formed by nanoparticles of 10–20-nm size (Fig. 3c). The nanoparticles of larger size, ~100 nm, are characteristic for the samples with higher concentration of dopants, La_0.8_Eu_0.1_Ca_0.1_VO_4_. Most of them have polyhedral shapes with clearly defined edges and angles between them (Fig. 3d). The particles with the observed polyhedral shapes have to be assigned to the crystal system of lower symmetry. Thus, most likely, they should be regarded as a manifestation of the monoclinic phase of the studied samples. The next increase of the Eu^3+^ and Ca^2+^ dopant concentrations also generates particles of ~100-nm sizes (Fig. 3e). Despite of the poor quality of this image, it is possible to conclude that the shapes of the particles are closer to the cylinders and rods.The same shapes are observed for the samples of the highest dopant concentration, La_0.6_Eu_0.2_Ca_0.2_VO_4_ (Fig. 3f). However, it should be noted that size of the particles is smaller for these samples; it is preferably 30–60 nm.

Thus, we can say that the change of the powder samples’ composition is accompanied by the changes in the size and morphology of their constituent particles. These changes are consistent with the XRD data on the structure and phase composition of the samples. Therefore, the SEM data can be interpreted as indicating that the samples of higher dopant concentration are mixtures of monoclinic and tetragonal phases and the content of the latter increases when the content of europium and calcium ions increases.

Chemical element analysis was performed for 3–5 various agglomerates or grains for each sample using SEM tools. Monitoring area from which information about content of atoms have been taken was larger than e-beam size, and it was near (30–40) × (30–40) μm. We have found that La, Eu, Ca, V, and O are the main components of the samples. In certain cases, some quantity of carbon atoms revealed that obvious occurred when edge zone of grains or grains of small thickness were monitored. (Powder samples were fixed on special carbon-covered scotch tape for SEM measurements.) Average data are accumulated in Table 1.

**Table 1:** Calculated starting composition of the La, Ca, and Eu atoms in the samples under synthesis, average content of atoms (in at.%), and evaluated composition of the synthesized samples

Starting composition	La	Eu	Ca	Evaluated composition
La_0.8_Ca_0.2_	41.2	–	9.4	La_0.81_Ca_0.19_
La_0.9_Eu_0.05_Ca_0.05_	26.60	0.73	1.76	La_0.92_Eu_0.02_Ca_0.06_
La_0.8_Eu_0.10_Ca_0.10_	20.81	2.34	1.11	La_0.86_Eu_0.10_Ca_0.04_
La_0.7_Eu_0.15_Ca_0.15_	12.30	3.30	1.73	La_0.71_Eu_0.19_Ca_0.10_
La_0.6_Eu_0.20_Ca_0.20_	12.88	7.07	2.97	La_0.56_Eu_0.31_Ca_0.13_

Analysis of data presented in Table 1 gives possibility to note the next.

1. Data of the SEM experiments on the contents of the La^3+^ ions in the synthesized samples (first column of the Table 1) are quite closed to the expected compositions (see the far right column of the Table 1).
2. If we take the total content of the Eu^3+^ and Ca^2+^ ions, it is easy to see that the experimentally measured data are quite close to the expected compositions. However, it is easy to see that the predicted simultaneous fourfold increase of ions Eu^3+^ and Ca^2+^ content (from 0.05 up to 0.20 at.%) is not implemented for the actual synthesized samples. We can observe when the content of the Eu^3+^ ions increases by ~10 times, the content of the Ca^2+^ ions increases only by ~2–3 times. This is due both to specific of the samples (heterogeneity of sizes, shapes, and thickness of grains of powder, etc.) and features of the method (a significant error for light elements, the effect of electron beam on oxide surfaces, etc.). Nevertheless, this result should be taken into account when describing other properties of the synthesized compounds. First of all, it concerns their luminescent properties. To avoid misunderstanding, we will not change following designation of samples noted in the “Synthesis” and “X-ray Diffraction” sections.

### IR Spectroscopy

**Fig. 4:**
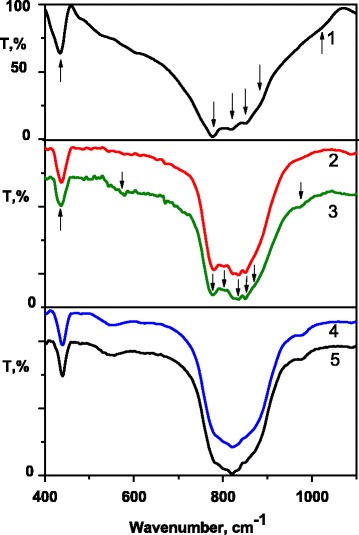
IR absorption spectra of the LaVO4 (*1*), La0.9Ca0.1VO4 (*2*), La0.8Ca0.2VO4 (*3*), La0.8Eu0.1Ca0.1VO4 (*4*), and La0.6Eu0.2Ca0.2VO4 (*5*)

IR spectroscopy study of the La_1 − *x*_Ca_*x*_VO_4_ and La_1 − *x* − *y*_Eu_*y*_Ca_*x*_VO_4_ (0 ≤ *x*, *y* ≤ 0.2) samples was performed to confirm their structure and composition. The view of the IR spectra in the range of 400–1100 cm^−1^ (Fig. 4) is typical for the LaVO_4_ IR absorption spectra previously measured in this range [28–30]. These spectra are also very similar to the IR spectra of other lanthanide orthovanadates LnVO_4_ (Ln = Y, Ce−Yb) [28, 31], multi-metal orthovanadates such as M_3_Ln(XO_4_)_2_ and M_2_M**′**Ln(XO_4_)_2_ (M, M**′** = Na, K, Rb) [32] and vanadates of the M**′′**_3_V_2_O_8_ (M**′′** = Mg, Ca, Sr, Ba, and Zn) type [33–35]. Namely, the spectra are similar to all of compounds; those lattices are built by isolated tetrahedral VO_4_^3−^ molecular groups.

It is well known that the bending (ν_2_ and ν_4_) and stretching vibrations (ν_1_ and ν_3_) of O–V–O bonds of the VO_4_^3−^ anion form IR absorption spectra of various orthovanadates in the range 400–700 and 700–1100 cm^−1^, respectively [36, 37]. In fact, measured by us, spectrum of the undoped LaVO_4_ contains separated weak bands located in the range 400–700 cm^−1^, while stronger wide band lies in the range 700–1100 cm^−1^ (Fig. 4, curve 1). Sharp peak at 434 cm^−1^ and blurred band centered near 550 cm^−1^ compose IR spectra of this sample in the former range. The main peak at ~778 cm^−1^ and three strongly overlapped other ones located at ~820, 850, and 880 cm^−1^ form wide band (Fig. 4, curve 1). (All the mentioned peaks are marked by arrows close to the curve 1 in Fig. 4, and their positions are in Table 2).

**Table 2:** IR peaks positions (in cm^−1^) and their attributions to vibration modes in the VO_4_^3−^ vanadate groups

LaVO_4_	La_0.9_Ca_0.1_VO_4_	La_0.8_Ca_0.2_VO_4_	La_0.8_Eu_0.1_Ca_0.1_VO_4_	La_0.6_Eu_0.2_Ca_0.2_VO_4_	Vibration modes
434	438	436	439	440	ν_4_
		570	553	553	
778	780	777	783	780	
	802	801	802	801	
				811	ν_3_
820	822	824	821	820	
850	836; 850	835; 850	835; 854	836; 852	
880	874	875	875	874	ν_1_
1026	976	975	918; 978	975	

When LaVO_4_ is doped by the Ca^2+^ ions, the shapes of spectral bands and peaks positions are changed, but these changes are not as striking as one could expect, but we should note that composition of peaks changes. As a result, six peaks marked by arrows can be found on the curves 2 and 3 of Fig. 4 in the range of wide absorption band.

If the Eu^3+^ ions are added to the La_1 − *x*_Ca_*x*_VO_4_ compounds, then band of stretching vibrations becomes more complicated and its components are more overlapped (Fig. 4, curves 4 and 5). Thus, six or seven peaks can be distinguished there (see arrows), and shape and structure of the band become very similar to ones previously published on IR absorption spectra of the LaVO_4_ containing Eu^3+^ ions [31, 36]. (The peaks positions of all measured IR absorption bands are accumulated in Table 2.)

The exact assignment of spectral components to a certain type of modes is difficult, especially that concerns stretching vibration range. Due to the low C_s_ symmetry of the VO_4_^3−^ molecular anion in monoclinic LaVO_4_, four IR lines lying close to each other can appear in this range of spectra. Our experimental findings are in good agreement with noted above theoretical prediction. Mentioned fact causes strong overlapping of the lines, that is why they are revealed as one complex wide band. Doping with calcium ions causes a distortion of certain amount of vanadates groups. As a result, additional peaks appear in the spectrum. Besides, as we have shown above, all co-doped samples under our study are a mixture of monoclinic and tetragonal LaVO_4_ phases and contribution of the phases depends on the dopant concentration. The VO_4_^3−^ molecular groups possess D_2d_ symmetry in tetragonal LaVO_4_. So, two lines of E_u_ and A_2u_ symmetry can be found in the range of stretching vibrations if morphology of the VO_4_^3−^ groups corresponds to ideal tetragonal lattice structure. Really, neighbor environment and symmetry of some VO_4_^3−^ groups in tetragonal lattice are also deformed by Ca^2+^ ions, and all of VO_4_^3−^ internal vibrations can occur in the IR spectra. As previously have been reported, the calculated [38] and measured [31] peak positions of the IR absorption lines for tetragonal LaVO_4_ are close to those for monoclinic LaVO_4_. Thus, the eight lines can form the range of stretching vibrations, we suppose. Change of the phase composition leads to mentioned changes of the shape of the IR bands and their positions. Regarding published theoretical and experimental data, a possible assignment of all measured features was made (Table 2). Additional studies are necessary in order to clarify the origin of the bands that are in the ranges 500–700 and 900–1000 cm^−1^. We can only note that similar features were previously observed in the IR absorption spectra of some Ca containing orthovanadates [33, 34].

In any cases, we are able to state that observed IR spectra confirm that anionic sub-lattice of studied vanadates is built by VO_4_^3−^ molecular anions.

### Luminescent Spectroscopy

**Fig. 5:**
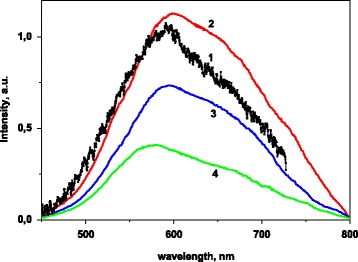
Emission spectra of the La_1 − *x*_Ca_*x*_VO_4_ samples, λ_ex_ = 310 (*1*–*3*) and 290 nm (*4*); x = 0 (*1*), 0.2 (*2*), and 0.1 (*3*, *4*), *T* = 77 K

Emission of the La_1 − *x*_Ca_*x*_VO_4_ samples is observed in a wide spectral range from 400 to 800 nm. The observed bands are complex. The photoluminescence (PL) spectra contain two wide and strongly overlapped components at position of their maximum near ~570 and ~630 nm (Fig. 5). The band positions and structure of the spectra are similar to those described previously as for undoped nanosized LaVO_4_ and Ba_3_V_2_O_8_ compounds, as well as for activated Sr_2.91_V_2_O_8_:0.06Eu^3+^, Ca_2_NaMg_2_V_3_O_12_:Eu^3+^, and Na_2_LnMg_2_V_3_O_12_ vanadate phosphors [17, 21, 39, 40]. The observed bands can be assigned to electron transitions in the VO_4_^3−^ groups; those are constituents of mentioned compounds. The charge transfer transitions of 3d orbitals electron of the V^5+^ ion to the 2p orbital electron of the O^2−^ ion inside of the VO_4_^3−^ groups are the nature of this luminescence [40, 41]. According to energy scheme of the vanadate tetrahedron, emission bands at 570 and 630 nm are connected respectively with ^3^T_2_ and ^3^T_1_ → ^1^A_1_ electron transitions in the VO_4_^3−^ groups [39–41]. These transitions are forbidden in the ideal T_d_ symmetry of the VO_4_^3−^ group. Distortion of the VO_4_^3−^ tetrahedrons from the ideal symmetry in the crystal lattice of LaVO_4_ enhances the spin–orbit interaction and makes these transitions partially allowed [42]. As we noted above, in the monoclinic phase of the La_1 − *x*_Ca_*x*_VO_4_ samples, the VO_4_^3−^ groups have distorted symmetry. Besides, some of these groups are affected by Ca^2+^ ions. The VO_4_^3−^ group also is of lower symmetry than T_d_ in tetragonal La_1 − *x* − *y*_Eu_*y*_Ca_*x*_VO_4_ samples. Thus, we have all the reasons to assign the observed 570 and 630 nm bands to the ^3^T_2_ and ^3^T_1_ → ^1^A_1_ electron transitions in the VO_4_^3−^ groups.

**Fig. 6:**
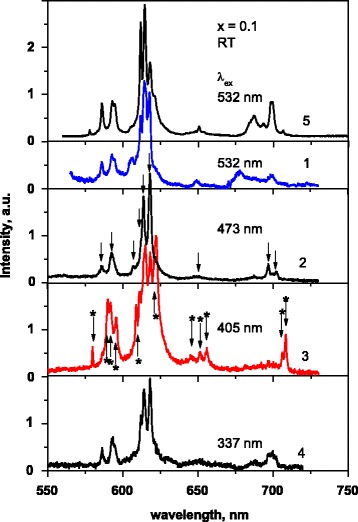
Emission spectra of the La0.8Eu0.1Ca0.1VO4 (*1*–*4*) and La0.9Eu0.1VO4 (*5*) samples at λex = 532 (*1*, *5*), 473 (*2*), 405 (*3*), and 337 nm (*4*)

**Fig. 7:**
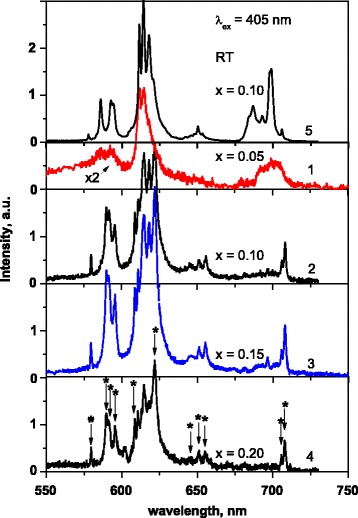
Emission spectra of the La_1 − *x* − *y*_Eu_*y*_Ca_x_VO_4_ (*1*–*4*) and La_1 − *x*_Eu_*x*_VO_4_ (*5*) samples at λ_ex_ = 405 nm; *x*, *y* = 0.05 (*1*), 0.1 (*2*, *5*), 0.15 (*3*), 0.2 (*4*)

Emission of the La_1 − *x* − *y*_Eu_*y*_Ca_*x*_VO_4_ samples is observed in the 550–730 nm spectral range and consists of narrow spectral lines, and undoubtedly, they are caused by radiation transitions in Eu^3+^ ions (Figs. 6 and 7). The emission intensity increases if content of dopants increases in the range (0 ≤ x ≤ 0.15) (Fig. 7).

**Fig. 8:**
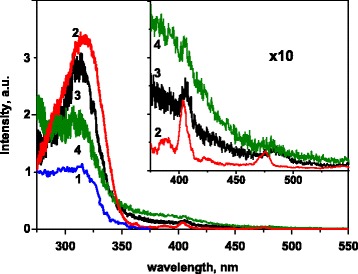
Excitation spectra of the LaVO4 (*1*), La0.9Eu0.1VO4 (*2*), and La0.6Eu0.2Ca0.2VO4 (*3*, *4*) samples at λem = 550 (*1*), 614 (*2*, *3*), and 622 nm (*4*); *T* = 77 K (*1*), T = RT (*2*–*4*)

Excitation spectra of all the synthesized samples are typical for luminescence excitation spectra of undoped and doped with Eu^3+^ ions LaVO_4_ [20–23, 42–44]. They contain wide band with peak position at about 320 nm (Fig. 8). Also, two narrow weak peaks at ~405 and ~475 nm are presented in the spectra of the Eu-containing samples.

Structure of the emission spectra depends on the excitation wave length, λ_ex_ (Fig. 6), and on the concentration of the Ca^2+^ and Eu^3+^ dopants (Fig. 7). For the La_0.8_Eu_0_._1_Ca_0.1_VO_4_ sample, which is just a mixture of monoclinic and tetragonal phases (see Fig. 2), the PL lines at 586, 592.5, 607, 611, 614, 618, 649, 697, and 702 nm (marked by arrows at Fig. 6, curve 2) are observed at all applied λ_ex_. (*We call this set of the lines as first set*.) Note also that the ratio of intensity of the lines at 611, 614, and 618 nm changes if λ_ex_ varies. Besides, intensive lines at 580, 590, 591.7, 595.6, 608.7, and 622 and lines near 645, 651, 655, 706, and 708 nm (marked by arrows with asterisk at Fig. 6, curve 3, and at Fig. 7, curve 4) are distinctively observed in the spectra measured at λ_ex_ = 405 nm. (*We call this set of the lines as second set.*) So, the measured emission spectra are the superposition of two sets of luminescence lines. Since we have shown above that the sample La_0.8_Eu_0_._1_Ca_0.1_VO_4_ is just a mixture of two crystalline phases, we have first to suppose that these sets are related with emission of Eu^3+^ centers in monoclinic and tetragonal phases.

With the intent to clarify this assumption, we performed study of the luminescence spectra dependence on the samples composition at the same excitation wave length, λ_ex_ = 405 nm (Fig. 7). We found that the emission spectra of the samples where *x* = 0.1 and 0.15 are very close to each other (Fig. 7, curves 3 and 4). This finding agrees with the statement that these samples are the mixture of two phases. Tetragonal phase dominates in the La_0.6_Eu_0.2_Ca_0.2_VO_4_ sample, and we see that *second set of the luminescence lines also* dominates in the spectrum of this sample, but we see also that mentioned before line at 618 nm is vanished in this spectrum (Fig. 7, curve 4). When observe the emission spectrum of the La_0.9_Eu_0.05_Ca_0.05_VO_4_ (Fig. 7, curve 1), we see that *first set of lines* mainly reveals in the spectrum (Fig. 7, curve 1). As the La_0.9_Eu_0.05_Ca_0.05_VO_4_ sample is monoclinic (see Fig. 2), we should relate the *first set* of lines to emission of Eu^3+^ ions in monoclinic crystal phase. At the same time, we note that line at 618 nm is also vanished in this spectrum. So, we concluded that not only competition between content of the crystal phases influence luminescence behavior. Let us discuss the question in detail.

The observed narrow PL lines are caused by the ^5^D_0_ → ^7^F_J_ (J = 0, 1, 2, 3, 4) electron radiation transitions in the inner *4f* ^*n*^ shell of the Eu^3+^ ions. The ^7^F_J_ energy levels may be split in the crystal field on the 2j + 1 sub-levels, but some of possible transitions can be forbidden by the symmetry rules [45]. If the Eu^3+^ ions occupy the site of the La^3+^ ions in tetragonal LaVO_4_ crystal lattice (I41/amd space group), they have D_2d_ site symmetry. In such a case, group theory predicts no luminescence peaks from ^5^D_0_ → ^7^F_0_ transition, two lines from the ^5^D_0_ → ^7^F_1_ and three lines from the ^5^D_0_ → ^7^F_2_ transitions. If the Eu^3+^ ions occupy the site of the La^3+^ ions in monoclinic LaVO_4_ crystal lattice (P2_1_/n space group), they have C_1_ site symmetry. In this case, group theory predicts manifestation all of possible transitions: one luminescence peak from the ^5^D_0_ → ^7^F_0_ transition, three lines from ^5^D_0_ → ^7^F_1_ and five lines from the ^5^D_0_ → ^7^F_2_ transitions [44].

So, it was not surprising that accounting the spectra in Fig. 6 in sum, we have found one line for the ^5^D_0_ → ^7^F_0_ (spectral range 570–585 nm), five lines (586, 592.5, 590, 591.7, 595.6 nm) for the ^5^D_0_ → ^7^F_1_ (spectral range 585–600 nm), and seven lines (607, 614, 618, 608.7, 611, 614, 622 nm) for ^5^D_0_ → ^7^F_2_ transitions (spectral range 600–650 nm), as this figure shows the luminescence spectra of the sample which is mixture of the monoclinic and tetragonal phases. This statement also concerns the spectra 2 and 3 in Fig. 7, as they also represent luminescence of the mixture, *x* = 0.1 and 0.15, respectively. It was surprising that we see intensive line from the ^5^D_0_ → ^7^F_0_ transition for the La_0.6_Eu_0.2_Ca_0.2_VO_4_ sample which represents only tetragonal crystal structure (Fig. 7, curve 4). Moreover, we see that the spectrum of the monoclinic La_0.9_Eu_0.05_Ca_0.05_VO_4_ sample (Fig. 7, curve 1) is lesser complicated than it might be expected to this structure. At the same time, it is important that luminescence intensity of these samples is lower if compared to intensity for other samples shown in this Figure. As we go from spectrum 1 to spectrum 4 in Fig. 7, the contribution of monoclinic phase decreases. These results mean that other factors, not only crystal structure, significantly determine luminescence behavior of the samples under study. Thus, we supposed that as shown in Figs. 6 and 7, curves 2–4, spectral transformations are mainly related to luminescence behavior of tetragonal phase, especially to role of Ca^2+^ cations, as their concentration increases by 2–3 times when gone from *x* = 0.1 to 0.2 (see the “Morphology and Chemical Element analysis” section).

In fact, obtained results mean that at least two of different type luminescence centers formed by the ions Eu^3+^ in the sample of tetragonal structure contribute to the PL spectra. We ascribed the abovementioned spectral lines to emission of type I (“first set” of lines) and type II of the centers (“second set” of lines). Emission line from the ^5^D_0_ → ^7^F_0_ transition (580 nm) is observed only for the type II centers, and the most intensive lines of the ^5^D_0_ → ^7^F_2_ and ^5^D_0_ → ^7^F_4_ transitions for these centers are located at 622 and 708 nm, respectively. For type I centers, the most intensive lines are located at 614 and 697 nm. These spectral features allow us to do assumption that types I and II of centers are characterized by different symmetry of the Eu^3+^ ions environment: the type I centers are characterized by higher site symmetry and the type II centers are characterized by lower site symmetry in the crystal lattices [45]. We supposed that type I centers are formed by the Eu^3+^ ions in their regular positions in the crystal lattice (D_2d_ symmetry).

As for the type II centers, we suppose that they can be formed by Eu^3+^ ions disturbed by defects generated as a result of Ca^2+^ cations incorporation into LaVO_4_ crystal lattice. To confirm this supposition, we performed additive experimental study of the sample that does not contain Ca^2+^ ions, namely, La_0.9_Eu_0.1_VO_4_ composition of tetragonal structure [14, 23] (Figs. 6 and 7, curves 5). Taking into account that above noted lines of type II centers (e.g., lines at 622 and 708 nm) were not observed for the Ca^2+^-free sample (Fig. 7, curve 5), we concluded that the most possible origin of the type II centers is the Eu^3+^ ions disturbed by Ca-induced defects. In the case of the doped with calcium LaVO_4_ lattice, the Ca^2+^ dopant has to replace the La^3+^ ions. Then, effective “−1” charge arises. Charge compensation of mentioned −1 charge is needed, as the crystal has to be electro-neutral. This compensation can be achieved via formation of one oxygen vacancy, [V_O_]^2+^, on each two Ca^2+^/La^3+^ replacements. Thus, for the Ca^2+^ dopants arrangement in the LaVO_4_ crystal lattice, we should expect that some of the Eu^3+^ ions are under the effect of both neighbor Ca^2+^ cations and oxygen vacancy. Thus, we can assume that such Eu^3+^ ions are just the type II of the luminescence centers, and their local symmetry is lower if comparing to symmetry of the type I centers. Taking into account that ^5^D_0_ → ^7^F_0_ transition emission line (near 580 nm) is observed in the spectra (Figs. 6 and 7), the symmetry of type II centers may be C_2_ or C_s_ [44, 45]. The C_2_ symmetry of the Eu^3+^ surrounding in the I41/amd space group of orthovanadates can be achieved only by equal distortions of two oxygen positions. Therefore, we assume that case of the C_s_ symmetry of the Eu^3+^ surrounding at the type II centers is more probable.

Excitation spectra measured in the emission lines corresponded to different types of centers (614 and 622 nm for types I and II centers, respectively) confirmed made above description, as these spectra revealed some differences (Fig. 8). The wide band at 320 nm is more intensive in the excitation spectra of type I centers. The 320-nm band is also observed in the spectra of the La_0.9_Eu_0.1_VO_4_ sample (Fig. 8, curve 2). This band is caused by electron transitions in the vanadate VO_4_^3−^ groups. Therefore, the type I centers are better excited through the matrix than type II centers, that confirms our assumptions about relation of the type I centers with the Eu^3+^ ions in the regular positions in the crystal lattice.

**Fig. 9:**
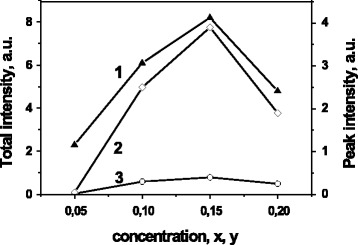
Dependencies of the La_1 − *x* − *y*_Eu_*y*_Ca_*x*_VO_4_ luminescence intensity on dopant concentration *x*; total emission (*1*) and 622 nm peak intensity (*2*, *3*); λex = 405 (*1*, *2*) and 473 nm (*3*)

Total emission intensity of the La_1 − *x* − *y*_Eu_*y*_Ca_*x*_VO_4_ samples have shown essential increasing when dopant concentration increases up to *x*, *y* = 0.15 (Fig. 9, curve 1). Similar dependence reveal peak intensity of the 614 and 622 nm lines caused by luminescence of types I and II centers, respectively (Fig. 9, curve 2 and 3), but we see that main contribution to luminescence enhance is related with emission of type II of centers. Therefore, spectral behavior of the type II luminescence centers is not only single manifestation of the Ca^2+^ cations influence on the La_1 − *x* − *y*_Eu_*y*_Ca_*x*_VO_4_ emission. The Ca^2+^ doping enhances also efficiency of this type of luminescence centers.

As for quenching of the luminescence intensity at *x*, *y* > 0.15, it should be noted that several mechanisms may be responsible for this. First, it is concentration quenching related with Eu^3+^ content. Then, as we described in the “Morphology and Chemical Element Analysis” section, the sizes of particle decreases for the La_0.6_Eu_0.2_Ca_0.2_VO_4_ sample. So, the surface quenching can be important mechanism in this case.

Surely, made assumption about role of the Ca^2+^ ions in luminescence of the lanthanum vanadate, LaVO_4_, co-doped with Eu^3+^ and Ca^2+^ ions requires additional study, and we plan to carry out it in the future.

## Conclusions

The La_1 − *x*_Ca_*x*_VO_4_ and La_1 − *x* − *y*_Eu_*y*_Ca_*x*_VO_4_ (0 ≤ *x*, *y* ≤ 0.2) micro/nanosized powders were prepared by aqueous nitrate–citrate sol–gel synthesis. Phase composition of the sample depends on the *x* and *y* values.

The La_0.9_Ca_0.1_VO_4_ is crystallized in monoclinic structure up to the *x* = 0.1.

The La_0.9_Eu_0.05_Ca_0.05_VO_4_ sample was also attributed to the monoclinic structure.

Increasing concentration of europium, Eu^3+^, and calcium ions, Ca^2+^, in La_1 − *x* − *y*_Eu_*y*_Ca_*x*_VO_4_ solid solutions leads to the change of the crystal structure, and subsequently, stabilization of the tetragonal phase takes place.

Phase transformation and especially Ca^2+^ ions influence IR spectroscopy and luminescence behavior of studied compounds, as Ca^2+^ ions impact both on VO_4_^3−^ molecular groups and La^3+^ and Eu^3+^ ions.

Emission of the La_1 − *x*_Ca_*x*_VO_4_ samples consists of wide bands in the 450–800 nm spectral range. These bands were ascribed to electron transitions in the distorted VO_4_^3−^ vanadate groups.

Emission of the La_1 − *x* − *y*_Eu_*y*_Ca_*x*_VO_4_ samples is observed in the 550–730 nm spectral range, and it consists of narrow spectral lines. These lines are caused by the ^5^D_0_ → ^7^F_J_ electron transitions in the Eu^3+^ ions. The Eu^3+^ ions form two types of emission centers in the samples under study. The assumption was made that type I centers are formed by the Eu^3+^ ions in their regular positions in the crystal lattice, while the type II centers have complex structure and they consists of Eu^3+^ ions, Ca^2+^ cations, and oxygen vacancies.

## Abbreviations

IR: Infrared
PL: Photoluminescence
RE: Rare earth
SEM: Scanning electron microscopy
XRD: X-ray diffraction
